# Genetically encoded biosensors of metabolic function for the study of neurodegeneration, a review and perspective

**DOI:** 10.1117/1.NPh.12.S2.S22805

**Published:** 2025-09-04

**Authors:** Minglei Zhao, Saman Behboudi Tanourlouee, Sean McCracken, Philip R. Williams

**Affiliations:** a Washington University School of Medicine, John F. Hardesty, MD Department of Ophthalmology and Visual Sciences, St. Louis, Missouri, United States; b Washington University School of Medicine, Department of Neuroscience, St. Louis, Missouri, United States; c Washington University School of Medicine, Hope Center for Neurological Disorders, St. Louis, Missouri, United States

**Keywords:** genetically encoded biosensors, metabolism, neurodegeneration

## Abstract

Nervous system tissue is the most metabolically active in the body and neurons are the primary consumers of oxygen and metabolites in nervous tissue. Many processes support neuronal metabolism, and dysregulation of these processes or intrinsic neuronal metabolism is often tied to neurodegenerative diseases. While many techniques are available to query metabolic function and disease (e.g. Seahorse XF, histology, immunostaining), almost all of these approaches are destructive and few offer cellular resolution. However, genetically encoded biosensors can optically measure metabolic features in any tissue with optical access. Biosensors represent an approach to non-destructively monitor metabolic components and regulatory signaling repeatedly over time in intact tissues. In this review, we discuss the application of genetically encoded biosensors that measure metabolites and metabolic processes as applied to studies of neurodegeneration.

The brain is the most metabolically active organ in the body, consuming ∼20% of total oxygen and 25% of total glucose.^1^ The metabolic demand of the central nervous system highlights the critical need to maintain energy balance in neurons, the primary energy consumer in this tissue.^2^ Metabolic changes in neurons and glial cells are associated with neurodegenerative diseases.^3^^,^^4^ Such disruptions are closely tied to the onset of neurodegeneration,^5^ emphasizing the need for precise monitoring of key metabolites and regulatory pathways with cellular and temporal resoultion.^4^

Various methods to study metabolism in cells and tissues are available, including mass spectrometry, colorimetric assays, Seahorse XF analyzers, and histological techniques.^6^ Traditional techniques, such as immunostaining and Western blots, offer endpoint data but fail to capture dynamic processes.^2^ Although more invasive methods, such as electrode recordings, enable real-time measurements, they do not offer cellular resolution and risk damage or stress, making long-term studies of neural tissue challenging.^3^^–^^6^ Therefore, minimally invasive, high-resolution techniques are essential for tracking dynamic signaling in healthy and diseased neurons and neural cells to provide practical solutions to current challenges.^7^ Genetically encoded biosensors have emerged as powerful tools, offering nondestructive measurements with high spatial and temporal resolution.^8^^,^^9^ They enable real-time tracking of metabolic changes at the cellular and even subcellular level. Subcellular targeting (for example, organelle-targeted biosensors) further reveals the dynamics of key metabolites within and across discrete cellular compartments. Importantly, metabolism does not function in isolation as it is closely integrated with signaling pathways that regulate cell fate and stress responses. Biosensors can also allow researchers to measure signaling states with downstream cellular outcomes, including adaptation, growth, and survival. Although recent reviews have compiled an extensive compendium of genetically encoded biosensors and their properties,^8^^,^^9^ herein, we attempt to focus on biosensors applied at the intersection of cell metabolism and neurodegeneration.

## 1 Substrate Biosensors

Over the past decades, significant progress has been made toward the development of genetically encoded biosensors for real-time monitoring of metabolites *in vivo*. Biosensors have been designed, and in some cases are widely used, to measure key metabolites such as ATP, NAD(H), glucose, lactate, and pyruvate. This toolkit provides detailed spatial–temporal resolution from living samples at various stages of disease progression.^7^^,^^10^^,^^11^

Significant disturbances in cellular energy production and homeostasis play a key role in the development and progression of neurodegenerative diseases such as Alzheimer’s (AD), Parkinson’s (PD), and Huntington’s (HD).^2^^,^^3^^,^^5^ Degenerative metabolic decline is likely resultant from multiple compounding factors, including vascular disturbances, glial activation, and intrinsic defects of early degenerative neurons.^4^^,^^12^^–^^14^ The energetic failure in neurons is viewed as a mechanism contributing to neuronal death in degenerative conditions. As a result, metabolic interventions have been explored to provide supplemental or alternative energy sources to the CNS as potential treatments.^5^^,^^15^^–^^17^ Genetically encoded biosensors can measure essential metabolites to understand how cellular degeneration or resiliency is linked to energy demands.

### 1.1 ATP

Adenosine 5′-triphosphate (ATP) serves as the primary energy currency in living cells, playing a crucial role both as an energy source and a signaling molecule by modulating ion channels and activating signaling cascades.^18^^–^^20^ Mitochondrial dysfunction, commonly seen in neurodegenerative diseases such as AD, PD, and HD, often leads to a reduction in ATP.^21^^–^^23^ This shortage impairs neuronal energetic capacity, disrupts metabolism, increases vulnerability to oxidative stress, and accelerates neurodegeneration.^24^ Neurodegeneration can be slowed or reversed by restoring ATP homeostasis. For example, in PD, enhancing ATP levels through the supplementation of various substrates can protect neurons.^21^^,^^24^^,^^25^ In glaucoma, which is a leading cause of irreversible blindness, restoring mitochondrial transport to increase ATP production can prevent energy deficits and promote the survival of retinal ganglion cells.^26^ Monitoring ATP dynamics *in vivo* is thus critical for understanding the interplay between energy metabolism and cellular signaling processes in both physiological and pathological conditions.^22^ To this end, several genetically encoded ATP biosensors have been developed.

#### 1.1.1 ATeams

ATeam biosensors are Förster resonance energy transfer (FRET)-based and integrate the ATP-binding ε subunit of *Bacillus subtilis* $F_{0}F_{1}$-ATP synthase between mseCFP and mVenus. Four ATeam variants have been developed (ATeam1.03, ATeam1.03^YEMK^, ATeam3.10, and ATeam3.10^MGK^), with sensitivity to ATP $K_{d}$ ranges from 7.4  μM to 3.3 mM. ATeam1.03^YEMK^ is the most commonly used ATeam family member and has a 150% dynamic range that is centered around physiological intracellular ATP levels.^27^ ATeams have been heavily used to study the relationships between neuronal activity and metabolism^27^^–^^30^ but have also been applied to a number of neurodegeneration models.^26^^,^^31^^,^^32^ ATeam1.03^NL^, which is optimized for lower temperatures, was expressed in a *Drosophila* model of mitochondrial dysfunction, which causes developmental dendritic dystrophy to determine that mitochondrial *prel* mutation did not impair cellular ATP levels in L3 neurons but instead conferred cellular stress.^33^ Multiple ATeam variants have been applied to a streptozotocin model of diabetic neuropathy. In primary cultures of rat dorsal root ganglion neurons, diabetes induced lower resting ATP levels that were restored with treatments of insulin-like growth factor.^34^ *In vivo* imaging of ATP levels with ATeam in retinal ganglion cells, which degenerate in a mouse model of glaucoma, found that increased intraocular pressure causes reduced ATP levels that may be resultant from lower mitochondrial transport.^26^ Restoring mitochondrial transport by overexpression of disrupted-in-schizophrenia 1 (Disc1) protected retinal ganglion cells from degeneration.^26^

#### 1.1.2 iATPSnFRs

The single-wavelength ATP sensors, iATPSnFRs, were developed by incorporating circularly permuted superfolder GFP (cpSFGFP) into the ε subunit of $F_{0}F_{1}$-ATP synthase from *Bacillus PS3*. Although iATPSnFRs are pH sensitive and have a lower affinity for ATP (${\mathrm{EC}}_{50}∼50$ to 120  μM) compared with ATeams, they are particularly suitable for detecting ATP at cell surfaces with the cell membrane by adding targeting sequences in mammalian cells. These sensors enable intensity measurements with a dynamic range of ∼two-fold and have revealed metabolic heterogeneity at single synapses, likely reflecting local energy demands or distinct metabolic tuning at the synapse terminal.^35^^,^^36^ These experiments provide a foundation for studying synaptic vulnerability in neurodegeneration or activity-dependent plasticity at single synapse resolution.

#### 1.1.3 MaLions

Monitoring ATP level intensiometric turn-on (MaLions) are a family of spectrally diverse fluorescent ATP biosensors that consist of a split fluorescent protein (mApple MaLionR; citrine MaLionG; blue fluorescent protein MaLionB) flanking the ε subunit of $F_{0}F_{1}$-ATP synthase from *Bacillus sp. PS3*.^37^ ATP binding causes conformational changes in the split fluorescent protein, which increases fluorescence intensity. Their multicolored nature makes MaLions well-suited for simultaneously measuring ATP within multiple distinct cellular compartments or for performing measurements of different cellular pathways by pairing MaLions with other pathway biosensors. MaLions demonstrate differences in their $K_{d}$ (MaLionR: 0.34 mM; MaLionG: 1.1 mM; and MaLionB: 0.46 mM) and pH sensitivities (MaLionR, somewhat sensitive; MaLionG, sensitive to low pH; and MaLionB, insensitive), making them differentially appropriate for targeting specific cellular compartments.^37^ Last, MaLions possess robust dynamic ranges, making them applicable for *in vivo* experiments (MaLionR, 350%; MaLionG, 390%; MaLionB: 90%). MaLionG was used in primary neuronal cultures to investigate the mechanisms of age-related reductions in neuronal ATP levels. By expressing a cAMP response element–binding protein (CREB) inhibitory peptide within mitochondria and measuring ATP levels at the synapses with MaLionG targeted to the postsynaptic density by fusion to a fibronectin intrabody, it was discovered that mitochondrial CREB signaling is required to maintain postsynaptic ATP levels.^38^

#### 1.1.4 Perceval and PercevalHR

Perceval is an ATP/ADP ratio sensor created by inserting cpmVenus into an ATP-binding bacterial protein GlnK1. It responds specifically to the ATP/ADP ratio with an apparent half-maximal signal change ($K_{R}$) of ∼0.5 and has a dynamic range of ∼80%.^39^ Given that healthy mammalian ATP/ADP ratios vary widely, Perceval is often saturated and less sensitive at higher ratios.^39^ PercevalHR, an improved version, offers a dynamic range that is nearly fivefold greater than Perceval and a $K_{R}$ of ∼3.5, which better matches physiological ATP/ADP ratios and enables more reliable detection of ratios from 0.4 to 40.^40^ PercevalHR has been used to visualize the relationship between cellular energy states and the activation of $K^{+}$ channels.^40^ Using PercevalHR to measure ATP/ADP ratios in regenerating cortical neuron growth cones *in vitro* demonstrated that manipulations to increase mitochondrial transport boosted ATP/ADP levels and encouraged more extensive axon growth.^41^ A neuron culture model of Wolfram syndrome used PercevalHR to demonstrate reduced ATP/ADP ratios downstream of mitochondrial ${\mathrm{Ca}}^{2+}$ imbalance.^42^ *In vivo* PercevalHR imaging was used in a mouse model of multiple sclerosis, where reductions in ATP/ADP ratios decreased with disease progression and were lower in the same axon in regions close to inflammatory lesions.^43^ Dystrophic axons demonstrated lower ATP levels than healthy axons in the same inflammatory milieu, suggesting that metabolic dysfunction could drive axon degeneration in neuroinflammatory disease. Indeed, overexpression of TCA enzymes to restore ATP/ADP balance reversed disease progression, demonstrating the power of *in vivo* observational biosensor experiments.^43^

### 1.2 NAD(H)

NAD+ is a crucial metabolite involved in various cellular functions, including metabolic homeostasis, genomic stability, mitochondrial health, adaptive stress responses, and cell survival.^44^ It also acts as a critical redox cofactor.^45^ In neurodegenerative diseases such as AD, PD, HD, amyotrophic lateral sclerosis (ALS), and glaucoma, the dysregulation of NAD+ significantly disrupts energy balance and accelerates disease progression,^46^^–^^48^ whereas reductions in NAD+ levels are present in many neurodegenerative conditions.^49^^,^^50^ Strategies to boost or maintain NAD+ levels or its precursors appear promising in slowing the progression of neurodegeneration.^48^^,^^51^ In addition, the NADase-SARM1 has recently been discovered as an effector of axonal degeneration by depleting NAD+. Overexpressing the NAD+ synthesis enzyme NMNAT1 or inhibiting SARM activity can inhibit axonal degradation by preventing SARM1-mediated NAD+ depletion in a range of neurodegenerative models.^52^ Understanding these molecular and cellular mechanisms behind NAD+ metabolism offers potential new treatments for a variety of neurodegenerative diseases.

In all living cells, NADH is converted to NAD+ by the reduction of pyruvate to lactate when oxygen is limited and cells undergo anaerobic glycolysis. With sufficient oxygen, cytoplasmic NADH transfers its reducing equivalents to the mitochondrial matrix.^41^ This process allows NAD+ and NADH to interconvert and maintain balance without being consumed. The cytosolic NAD+/NADH redox balance is a key indicator of glycolysis and overall cellular metabolism.^53^ The deterioration of this balance and mitochondrial function is a critical factor in neurodegenerative diseases and aging.^46^

#### 1.2.1 NAD+/NADH biosensors Peredox and SoNar

Peredox is a biosensor designed to monitor the NAD+/NADH ratio. It consists of cpT-sapphire between two subunits of the NADH-sensitive T-Rex protein from *T. aquatics*. Peredox fluorescence increases with binding to NADH, whereas its signal decreases as NAD+ concentration increases and demonstrates a 150% dynamic range.^54^ *In vivo* imaging of Peredox in mouse cortical neurons demonstrated decreases in NADH levels after inhibition of the monocarboxylate transporter, and that NADH levels increased after physiological stimulation of the whiskers.^55^ In a neuronal culture model of Wolfram syndrome, Peredox was used to observe reduced NAD+/NADH ratios in Wolfram syndrome disease modeled neurons that displayed defective communication between endoplasmic reticulum and mitochondria.^42^

SoNar is a second-generation NAD+/NADH sensor developed by integrating cpYFP between residues 189 and 190 of a modified T-Rex monomer from *T. aquatics* without the DNA-binding domain. This excitation ratiometric sensor (420 and 485 nm) responds to changes in NADH and NAD+ with opposing fluorescence shifts. When used as an excitation ratiometric sensor, SoNar boasts a 1500% dynamic range, making it sensitive enough to study NAD redox states across subcellular compartments *in vivo* by fusion of different organellar targeting sequences.^56^^,^^57^ SoNar was also used to observe reduced NAD+/NADH ratios in Wolfram syndrome disease modeled.^42^

#### 1.2.2 NAD(H) levels FiNAD

The FiNAD sensor also features cpYFP inserted between residues 189 and 190 of the T-Rex monomer using short polypeptide linkers. FiNAD indicates the NAD+/AXP ratio, where AXP is the total pool of ATP and ADP. This sensor exhibits a sevenfold increase in fluorescence when excited at 485 nm in the presence of NAD+. FiNAD provides a rapid, sensitive, and specific real-time readout of NAD+ dynamics, but unlike Peredox and SoNAR, FiNAD has not yet been used *in vivo* broadly outside of its initial report, where it was applied to whole zebrafish embryos and mouse muscle tissue.^58^

### 1.3 Glucose

Glucose metabolism is an essential activity for neuronal function. In neurodegenerative diseases, glucose metabolism is often impaired, characterized by reduced cellular glucose levels, decreased glucose uptake, mitochondrial dysfunction, and diminished glycolytic flux.^59^ Such dysregulation is associated with a decreased expression of key glucose regulatory components such as glucose transporter 1 and 3 (GLUT1 and GLUT3) in neurons or brain tissue.^60^ Restoring glucose metabolism in hippocampal neurons through indoleamine 2,3-dioxygenase 1 (IDO1) inhibition can rescue cognitive function in AD pathology.^61^ Similarly, disrupted glucose metabolism plays a role in the pathogenesis of glaucoma, and limiting metabolic dysregulation by replenishing pyruvate or administering rapamycin protects from neurodegeneration.^16^ Therefore, comprehending the mechanisms of glucose metabolism in neurons is essential for developing effective treatments for neurodegenerative diseases.

#### 1.3.1 FLIPglu

The FLIPglu glucose sensors have been refined over multiple iterations and represent a robust toolbox for monitoring glucose dynamics. The original FLIPglu was developed by fusing the glucose/galactose-binding protein MglB (from *E. coli*) between a CFP and YFP FRET pair.^62^ One variant after optimization from FLIPglu, termed ${\mathrm{FLII}}^{12}\mathrm{Pglu}\text{-}600\mu$, displays a 58% dynamic range *in vitro* and an apparent $K_{D}$ of 600  μM.^63^ Further refinements led to the creation of ${\mathrm{FLII}}^{12}\mathrm{Pglu}\text{-}700\mu \delta 6$, which emerged as the top performer among new mutants, showing a $K_{D}$ of 660  μM and a dynamic response of 74%.^64^ These glucose sensors have proven effective for real-time glucose monitoring in astrocytes and neurons, where synaptic stimulation, inhibition of glucose transporters, and mitochondrial inhibition all induced significant reductions in glucose concentration.^10^^,^^65^

### 1.4 Lactate

Lactate, a byproduct of glycolysis, serves not only as a metabolic substrate but also as a signaling molecule.^66^ Lactate can serve as an energy substrate provided by glia to neurons, and under pathophysiological conditions, it can detrimentally modulate cellular functions.^67^^,^^68^ Altered lactate metabolism is a common feature in the progression of neurodegenerative diseases. Lactate levels appear to be elevated in AD,^66^^,^^69^^,^^70^ which may result from the downregulation of oxidative phosphorylation–related genes and impaired mitochondrial metabolism. Gene transcription can also be modulated by the lactylation of histone lysine groups, which promotes gene transcription through epigenetic changes.^70^^,^^71^ For example, lactate can modulate microglia energetics by lactylation of histone H4K12, affecting glycolysis that impacts metabolism in AD.^72^ The question of whether lactate is beneficial or detrimental in the progression of neurodegenerative diseases remains open. Further research is necessary to explore the dual effects of lactate as its impact likely depends on the dynamic changes in lactate levels *in vivo*.

#### 1.4.1 Laconic

The first genetically encoded FRET biosensor for lactate, laconic, is based on the bacterial lactate–sensitive transcription factor LldR inserted between a mTFP and Venus FRET pair and is also suitable for fluorescence lifetime imaging microscopy (FLIM) imaging.^73^ Laconic can quantify lactate across a broad physiological range, detecting lactate from 1  μM to 10 mM, but with a rather limited dynamic response range around 10% ΔR/R.^74^ Despite its limited dynamic range, laconic has been used to make critical observations in normally functioning and diseased nervous tissues. The role of lactate signaling and glial involvement in the neuronal metabolic responses to circuit stimulation and arousal state has been examined extensively using laconic.^55^^,^^75^^–^^78^ Regarding neurodegeneration, recent experiments with primary cultured neurons measured lactate levels with laconic in transected axons. In axons that were treated to induce axon regeneration, regenerating axon tips demonstrated higher levels of local glycolysis, which leads to higher lactate levels, and independently increasing axonal glycolysis could promote axon growth.^32^

#### 1.4.2 eLACCO1 and R-iLACCO1

The lactate sensor, eLACCO1.1, consists of circularly permuted GFP (cpGFP) inserted inside the TTHA0766 lactate-binding periplasmic protein.^79^ Directed evolution to enhance its performance resulted in eLACCO2.1 that demonstrates a Kd of 580  μM.^80^ The red fluorescent L-lactate biosensor, R-iLACCO1, was created by incorporating cpmApple into the LIdR protein lactate–binding protein. This biosensor family includes R-iLACCO1, R-iLACCO1.1, and R-iLACCO1.2 and demonstrates a range of $K_{D}$ values 74, 230, and 350  μM, respectively. The LACCO biosensor family has very robust dynamic ranges *in situ*, but measurements of cultured neurons and astrocytes, or of mouse neurons *in vivo*, show rather limited biosensor dynamics, although lactate elevations in response to circuit activity were apparent.^80^

### 1.5 Pyruvate

Pyruvate, the end product of aerobic glycolysis, is transported into mitochondria for oxidative metabolism, playing a crucial role in cellular energy processes. Abnormalities in pyruvate metabolism are observed in several major neurodegenerative disorders, including Leigh’s syndrome, AD, and PD.^81^ Supplemental pyruvate can protect cells from oxidative stress and inflammation in PD models.^82^ In other studies, inhibiting the mitochondrial pyruvate carrier (MPC), a protein mediating import of pyruvate to mitochondria, may help slow the disease progression through augmentation of autophagy and inhibition of neuroinflammation.^15^ Exogenous pyruvate has been used to protect neurons and improve behavioral outcomes in animal models of traumatic brain injury (TBI) and glaucoma.^16^^,^^83^ In clinical trials, a combination of pyruvate and nicotinamide (vitamin B3) significantly improved visual function in patients with open-angle glaucoma.^17^^,^^51^

#### 1.5.1 Pyronic

The pyruvate sensor Pyronic incorporates the *E. coli* transcriptional regulator PdhR between an mTFP and Venus FRET pair. Pyronic exhibits a dynamic range of ∼20% with an apparent Kd of ∼107  μM. Its response remains stable against pH fluctuations and is suitable for FLIM.^84^^,^^85^ MPC Inhibition led to the progressive accumulation of cytosolic pyruvate in cultured neurons.^85^

#### 1.5.2 PyronicSF

The second-generation PyronicSF inserts cpGFP into PdhR. PyronicSF is an excitation ratiometric sensor with two excitation peaks near 488 and 405 nm and an emission peak near 515 nm, but the fluorescence signal excited at 488 nm is typically used for readings. When used as an excitation ratiometric sensor, PyronicSF shows a 250% dynamic response to pyruvate with an apparent $K_{D}$ of 480  μM and demonstrates high selectivity for pyruvate without responding to similar metabolites.^86^ PyronicSF was used to measure the pyruvate level in cultured astrocytes and demonstrated that pyruvate was optimally maintained in the cytoplasm to allow MPC to maintain anaplerosis.^86^

## 2 Mitochondrial Targeted Biosensors

Mitochondria are central to aerobic metabolism and critical for ATP production in neurons. Aside from genetically encoded biosensors, mitochondrial potentiometric dyes have been used to evaluate mitochondrial function and health in neurons extensively *ex vivo*, using compounds such as rhodamine 123,^87^^–^^89^ or tetramethylrhodamine methylester.^90^^–^^92^ A detailed experimental guideline for monitoring changes in mitochondrial membrane potential in primary cortical neurons exposed to mitochondrial respiratory chain inhibitors has been well described^93^ and can be generalized to neurons *ex vivo* or *in vivo* in other neurodegenerative conditions. However, this section will focus on genetically encoded biosensors that examine mitochondrial traits beyond membrane potential and allow for quantitative metabolic measurements in defined cell populations.

To date, biosensors for ATP, NADH, pH, redox state, cAMP, calcium ions (${\mathrm{Ca}}^{2+}$), pyruvate, and zinc have been used in the matrix, intermembrane space, and the outer membrane region of mitochondria in both animal and plant cells.^94^ However, fewer have been applied specifically in neurons or other cells in the nervous system. In this section, we focus on genetically encoded biosensors targeted to mitochondria and their applications to neuroscience with an emphasis on neurodegeneration.

### 2.1 Mitochondrial ATP

As the end-product of the electron transport chain (ETC), ATP is synthesized within the mitochondrial matrix. The controlled release of ATP from the mitochondria causes a separation between the mitochondria and cytosol that helps to maintain the proton gradient necessary for ATP production through oxidative phosphorylation.^95^ Neurons have different metabolic demands depending on their morphological and electrophysiological properties that are altered in degenerative conditions. Understanding how mitochondria-specific ATP dynamics and levels are maintained or disturbed can help determine the mechanisms that underlie neuronal mitochondrial health and pathology. ATP biosensors targeted to the mitochondria have been implemented in neurons to determine interesting distinctions of mitochondrial ATP levels and dynamics.

#### 2.1.1 Mito-ATeam

Mitochondrial targeted ATeam (mito-ATeam), a FRET ratiometric ATP biosensor, has been used to observe mitochondrial ATP dynamics of individual nonneuronal cells extensively *in vitro*,^96^^–^^98^ as well as primary motoneurons *in vitro*^99^ and axons *in vivo*.^31^ Mito-ATeam was used to examine the importance of mRNA transport in axonal metabolic maintenance. The biosensor was expressed in cultured mouse motoneuron axons, and knockout of the mRNA transport protein *CLUH* induced mitochondrial ATP depletions specifically in the axonal growth cones but not the cell soma, which caused reduced growth cone size.^99^ Mito-ATeam was also used to evaluate peripheral nerve axons *in vivo*, where increases in mitochondrial ATP were observed after electrical activation. Importantly, different models of peripheral neuropathy, including inflammatory demyelination and Charcot-Marie-Tooth 2A disease, led to reductions in mito-ATP and an inability to respond to electrical stimulation.^31^ Together, these studies highlight the usage of Mito-ATeam to not just evaluate somatic mitochondria but also determine mitochondrial ATP dynamics in subcellular compartments of neurons, including growth cones and neurites.

#### 2.1.2 Mito-MaLions

MaLions biosensors (monitoring ATP level intensiometric turn-on indicators) consist of the ε subunit of bacterial ATP synthase, with cyclically permutated red, green, and blue fluorophores, which have been localized to cytosolic, mitochondrial, and nuclear compartments of cells.^37^ Being available in nonoverlapping spectral variants enables evaluation of ATP flux among compartments. A version of MaLions, smacATPi, showed different mitochondrial and cytosolic ATP responses to hypoxia in HEK cells.^100^ MaLions targeted to nonmitochondrial compartments have been used to spectrally multiplex different biosensors and different subcellular readouts^38^; however, MaLions have not yet been applied to neuronal mitochondria *in vivo*.

### 2.2 Mitochondrial NAD

Mitochondrial NAD+ dynamics are pivotal for cellular bioenergetics, especially in neurons, where metabolic demands can fluctuate significantly. Disruptions in mitochondrial NAD+ levels impact energy metabolism and can drive neurodegeneration by compromising mitochondrial functions, including ATP production and redox balance.^46^ Mitochondrial-targeted biosensors have emerged as effective tools for real-time monitoring of NAD concentrations and oxidation states.^101^ For instance, using HEK293T cells and a ratiometric NAD+ sensor, LigA-cpVenus, distinct patterns of NAD+ dynamics in response to sirtuin in PARP inhibition were observed between the mitochondria as compared with the cytosol.^102^ Another intensity-based biosensor that detects NAD(H) levels, i.e., Frex or FrexH, has been targeted to mitochondria to observe a strong elevation of NAD(H) in the mitochondria of HEK293FT cells after ETC inhibition.^103^ RexYFP, another intensity-based biosensor, has been targeted to mitochondria to measure NAD+/NAD(H) ratios in cultured HeLa and HEK293 cells.^104^ To the best of our knowledge, neuronal mitochondrial NAD+/NAD(H) dynamics have not been assessed with these techniques, and the capacity of these biosensors to provide spatial and temporal resolution of mitochondrial NAD+ or NAD(H) levels or ratios could significantly advance our understanding of neuronal energetics in health and disease.

### 2.3 Mitochondrial Pyruvate

Regulation of mitochondrial pyruvate dynamics is critical for balancing ATP production between glycolysis and oxidative phosphorylation. The ability of neurons to efficiently maintain mitochondrial pyruvate levels is vital during periods of increased energy demand.^105^ Disruptions in mitochondrial pyruvate transport can hinder energy metabolism and subsequently contribute to neurodegenerative processes by compromising neuronal health and function.^106^ Development of genetically encoded pyruvate biosensors has enabled real-time monitoring of pyruvate dynamics in both the cytosol and mitochondira.^86^

Mitochondrial pyruvate has been investigated with PyronicSF, a second-generation excitation ratiometric pyruvate biosensor. Mito-PyronicSF was used to examine primary cultured astrocytes and *Drosophila* brain explants.^84^^,^^86^ Comparing cytosolic and mitochondrially targeted PyronicSF demonstrated that astrocytes maintain pyruvate concentrations lower in their mitochondria than in their cytoplasm, which positions mitochondrial pyruvate import as a crucial regulatory point for the balance of glycolysis to oxidative phosphorylation.^84^ In *Drosophila* brain explants, mitochondrial pyruvate was less sensitive than cytosolic stores to increased pyruvate concentrations in the culture media, showing that the cytosol buffers excess pyruvate.^86^ Evaluation of pyruvate across intracellular compartments represents an exciting strategy for determining subcellular dynamics, and further investigation of biosensors in this fashion for neurodegenerative conditions is warranted.

### 2.4 Mitochondrial Reactive Oxidative Species

Mitochondrial reactive oxygen species (ROS) are byproducts of cellular respiration, primarily generated in the ETC during ATP production but can also accumulate in stressful conditions. Although low levels of ROS are important for cell signaling and homeostasis,^107^ neurodegeneration can cause excessive ROS production and oxidative stress that damages proteins, lipids, and DNA, leading to cell death if left unchecked.^108^ Genetically encoded ROS biosensors have been used to evaluate mitochondrial ROS in neurons. The most widely used fluorescent redox sensors belong to the redox-sensitive GFP (roGFP) family of biosensors, which are mostly selective to glutathione redox potential ($E_{\mathrm{GSH}}$)^109^ and have been used in a variety of neuronal imaging applications^110^ (for a complete list, see review from Kostyuk et al.).

In TH-mito-roGFP transgenic mice, the excitation ratiometric biosensor roGFP1 is targeted to mitochondria using a mitochondrial matrix targeting sequence and expressed specifically in monoaminergic neurons using a tyrosine hydroxylase promoter.^111^ Mitochondrial oxidative stress was observed in dopaminergic neurons with mito-roGFP-1 in whole cell slice recordings of *Park7* knockout mouse models of PD.^111^ Mito-roGFP has also been applied to *ex vivo* models of PD to determine mitochondrial oxidative stress occurs in dopaminergic neurons dependent on high levels of circuit activity^112^ and to identify genes that regulate oxidative stress in primary dopaminergic neuronal cultures.^113^ Mito-roGFP was also used to identify an increase in ROS in cultured spinal motor neurons affected by spinal muscular atrophy, which were accompanied by reduced mitochondrial transport and increased mitochondrial fragmentation.^114^

For more accurate temporal dynamics, roGFP2 has been fused to the oxidoreductase glutaredoxin 1 (Grx1) to more rapidly reduce the sensor back from its oxidized state.^115^ Mito-Grx1-roGFP2 was used to evaluate neurons in transgenic mice *in vivo* by driving expression with a neuronal enriched Thy1 promoter.^116^ *In vivo* imaging of axons in the mouse spinal cord determined that axon damage causes a rapid spike in intracellular ${\mathrm{Ca}}^{2+}$ that triggers mitochondrial ROS production.^116^ This same imaging approach was used to demonstrate that a similar mitochondrial ${\mathrm{Ca}}^{2+}$ overload and ROS stress occur in a mouse model of multiple sclerosis.^43^ An interesting combination of mito-ATeam and mito-roGFP was used to look at mito-ATP along with mito-ROS in mouse spinal cord axons *in vivo*. Spatial dynamics of ROS in axons found that demyelinating axons greatly increased ROS levels separate from ATP-dynamics, indicating a decoupling of mitochondrial ROS and ATP levels.^31^^,^^43^

### 2.5 Mitochondrial Calcium

Mitochondria buffer cytoplasmic ${\mathrm{Ca}}^{2+}$ and directly interact with the endoplasmic reticulum to serve as critical regulators of ${\mathrm{Ca}}^{2+}$ homeostasis. Importantly, ${\mathrm{Ca}}^{2+}$ dynamics also regulate the tricarboxylic acid cycle and ETC functions within the mitochondria. Mitochondrial ${\mathrm{Ca}}^{2+}$ dysregulation impinges on cellular energetics and drives neurodegeneration in many diseases.^117^^–^^123^ Thus, mitochondrial ${\mathrm{Ca}}^{2+}$ is a critical regulator of cellular energetics that can be perturbed during neurodegeneration. Fluorescent dyes, such as Fura-2 and Rhod-2, −5N, and –FF, have been historically used to examine intracellular mitochondrial ${\mathrm{Ca}}^{2+}$ dynamics.^124^^–^^126^ However, these dyes have poor cellular specificity and can be difficult to distinguish between mitochondria and cytosolic ${\mathrm{Ca}}^{2+}$.^127^ Numerous genetically encoded ${\mathrm{Ca}}^{2+}$ biosensors are available that overcome these weaknesses, many of which have been targeted to neuronal mitochondria.

Genetically encoded ${\mathrm{Ca}}^{2+}$ indicators (GECIs) allow for measurements of mitochondrial ${\mathrm{Ca}}^{2+}$ in living animals with high temporal and spatial resolution. Several GECIs have been developed using different fluorescent protein components and ${\mathrm{Ca}}^{2+}$-binding motifs to allow for a range of excitation/emission spectra, ${\mathrm{Ca}}^{2+}$-binding affinities, and temporal response characteristics (reviewed in Calvo-Rodriguez et al.).^128^ GECIs that have been used to measure mitochondrial ${\mathrm{Ca}}^{2+}$ in neurons and neurodegenerative diseases include different versions of GCaMP,^129^ R-GECO,^130^^–^^132^ and other less common fluorescent probes such as 2mtYC3.6,^133^ 4mtD3cpv,^134^ or R-Pericam mt.^135^ Although not exhaustive, here we highlight some applications of mitochondrially targeted ${\mathrm{Ca}}^{2+}$ biosensors with an emphasis on neurodegenerative models *in vivo.*

### 2.6 Intensiometric mito-Ca^2+^ Biosensors

The most common biosensors for measuring ${\mathrm{Ca}}^{2+}$ are the GCaMP family of GECIs built on a fusion of a cyclically permutated green fluorescent protein (or mRuby and mApple for RCamp and R-GECO, respectively), the ${\mathrm{Ca}}^{2+}$-binding domain calmodulin, and M13 peptide. Although GCaMP can be used as an excitation ratiometric biosensor, it is most commonly used as an intensity-based ${\mathrm{Ca}}^{2+}$ sensor with many versions available to evaluate ${\mathrm{Ca}}^{2+}$ dynamics with different temporal and spectral properties. In *Drosophila*, GCaMP3.0 was targeted to mitochondria with a targeting sequence corresponding to amino acids 1-71 of the dP32 N-terminus.^136^ In a *Drosophila* model of PD, dopaminergic neurons showed mitochondria ${\mathrm{Ca}}^{2+}$ dysregulation that was affected by altered ER-mitochondria contacts.^137^ In addition, a reduction in mitochondrial ${\mathrm{Ca}}^{2+}$ uptake, and an increase in ${\mathrm{Ca}}^{2+}$ efflux, was observed and shown to be beneficial for neurons across *ex vivo* *Drosophila* genetic models of PD, HD, AD, and frontotemporal dementia.^122^

In primary mouse hippocampal neurons, R-GECO was targeted to the mitochondria using two targeting sequences from cytochrome C oxidase VIII added to the N-terminus. These experiments demonstrated that dendritic mitochondria had transient mitochondrial ${\mathrm{Ca}}^{2+}$ elevations that were reduced when knocking out quality control proteins for mitochondrial protein import.^138^ Mito-R-GECO has also been expressed in the rodent brain by intracortical injections of an adeno-associated expression virus.^132^^,^^139^ Using this paradigm, mitochondrial ${\mathrm{Ca}}^{2+}$ uptake was correlated with higher firing rates of pyramidal neurons in slice preparations.^140^ *In vivo* imaging of mito-R-GECO in zebrafish sensory neurons was used to determine ${\mathrm{Ca}}^{2+}$ dynamics during axon degeneration, where axotomy increased mitochondrial ${\mathrm{Ca}}^{2+}$ after cytoplasmic ${\mathrm{Ca}}^{2+}$ influx just prior to fragmentation.^141^

### 2.7 FRET-Based GECIs

As neurodegenerative conditions progress over weeks, months, and years, intensity-based ${\mathrm{Ca}}^{2+}$ sensors are not optimal for chronic, comparative measurements of ${\mathrm{Ca}}^{2+}$ dynamics. Thus, ratiometric GECIs, often based on FRET, are more commonly used to study pathological ${\mathrm{Ca}}^{2+}$ dynamics. N33D1cpv measures ${\mathrm{Ca}}^{2+}$ on the outer surface of mitochondria and consists of a CFP and cpVenus FRET pair among the calmodulin ${\mathrm{Ca}}^{2+}$-binding domain that is localized to the mitochondrial outer membrane by an N-terminal fusion of the mitochondrial protein Tom20.^142^ When expressed in HeLa cells, ${\mathrm{Ca}}^{2+}$ hotspots from 1  μM to 200 to 300  μM were observed in response to histamine stimulation a limited area just outside of the mitochondria.^143^ A newer version of the N33D1cpv biosensor, i.e., N33D3cp, with a higher affinity for ${\mathrm{Ca}}^{2+}$ was expressed in HeLa cells and showed that IP3R channels participate in reticular ${\mathrm{Ca}}^{2+}$ leak toward mitochondria.^144^

Yellow cameleon 3.6 (YC3.6) is another FRET-based ${\mathrm{Ca}}^{2+}$ biosensor, which uses a CFP and YFP FRET pair flanking a calmodulin and M13 ${\mathrm{Ca}}^{2+}$-binding motif. The YC3.6 biosensor, which was localized to mitochondria with a cytochrome oxidase subunit eight localization sequence, was used to evaluate neuronal function *in vivo* in a mouse model of AD and identified that an increase in mito-${\mathrm{Ca}}^{2+}$ was associated with neuronal death.^133^ This study explicitly shows that mitochondrial ${\mathrm{Ca}}^{2+}$ influx correlates with neurodegeneration, which is a common hypothesis in the field and assumed to occur in many models of neurodegenerative disease (see reviews: Verma et al.; Muller et al.; Ryan et al.; Calvo-Rodriguez et al.).^128^^,^^145^^–^^147^ However, there are a few other studies that directly demonstrate that mitochondrial ${\mathrm{Ca}}^{2+}$ influx is specifically a driver of neuronal death after injury or in disease models. Therefore, mitochondrial ${\mathrm{Ca}}^{2+}$ biosensors applied to models of neurodegeneration, especially *in vivo*, would be promising for future research given the availability of biosensors and the importance of definitively elucidating the role of mitochondrial ${\mathrm{Ca}}^{2+}$ in neurodegeneration to develop therapeutic interventions.

## 3 Cell Signaling Pathways

Signaling pathways, involving receptors, ligands, transducers, messengers, and regulators, enable cells to adapt metabolism to changes in their environment.^148^ Although many signaling components, such as keystone kinases and transcription factors, have been identified, capturing real-time activity in neurons remains difficult. In the following section, we explore key signaling pathways implicated in neurodegenerative diseases. For each pathway, we discuss genetically encoded biosensors specifically designed to monitor their activity, emphasizing evidence from neurological contexts and their demonstrated efficiency and feasibility in both *in vitro* and *in vivo* settings.

### 3.1 AMP-Activated Protein Kinase (AMPK)

The AMPK signaling pathway regulates cellular energetic and homeostatic mechanisms through direct and indirect processes.^149^^–^^151^ AMPK can be activated by AMP accumulation in conditions where ATP and ADP are depleted and can also be activated by the ${\mathrm{Ca}}^{2+}$-dependent/calmodulin-dependent protein kinase. Once triggered, AMPK initiates a cellular energy conservation program that inhibits energy-consuming lipid and protein biosynthetic processes while enhancing catabolic pathways such as glucose uptake, collectively aiming to restore cellular ATP levels.^152^ AMPK has a complex and context-sensitive role in multiple neurodegenerative conditions, including AD, glaucoma, and ALS.^153^^,^^154^^,^^156^ Activating AMPK can safeguard neurons from neurodegeneration by enhancing energy production through glycolysis and oxidative respiration. However, excessive or prolonged activation may cause harm *via* energy depletion and disrupted metabolic balance.^155^^–^^157^

#### 3.1.1 ABKAR

AMPK/BRSK activity reporter (ABKAR) is a second-generation FRET-based biosensor that monitors the activities of two critical kinases, AMPK and BR serine/threonine kinase (BRSK). ABKAR consists of an AMPK substrate peptide that interacts with a fork head–associated 1 domain (FHA1) between a Cerulean3 and cpVenusE172 FRET pair. The sensor demonstrates a nearly 40% dynamic range, which represents a twofold enhancement in dynamic range compared with the first-generation AMPKAR.^158^^,^^159^ ABKAR was tested *in vitro* in Cos7, HeLa, and HEK293T cell lines, as well as in primary rat and mouse hippocampal neurons by applying ionomycin or 2-deoxyglucose (2-DG).^160^ Addition of 2-DG led to a 10% to 20% increase in ABKAR activity in primary hippocampal neurons by 10 min after administration.^160^

### 3.2 Mechanistic Target of Rapamycin (mTOR)

When nutrients abound, mTOR expedites protein and lipid synthesis, thereby supporting cell growth and proliferation while inhibiting degradative pathways.^161^^–^^163^ In numerous neurodegenerative disorders, mTOR also plays a role in activating autophagy to clear cytotoxic protein aggregates, such as tau and amyloid-β in AD. Inhibiting mTOR benefits AD, PD, and HD by reducing levels of Aβ, α-synuclein, and mutant huntingtin.^164^^–^^166^

#### 3.2.1 AIMTOR

Activity indicator of mTOR (AIMTOR) uses bioluminescent resonance energy transfer (BRET) to monitor mTOR activity. The biosensor includes a mTORC1 substrate peptide from Unc-51, such as autophagy activating kinase 1, and a WW ligand–binding domain positioned between a nano-luciferase and the yellow YPET fluorescent protein.^167^ Upon phosphorylation by mTOR, the binding of the WW domain to the phosphorylated residue induces a conformational shift, bringing the donor and acceptor into closer proximity and then enhancing the BRET signal. AIMTOR demonstrates a BRET dynamic range of ∼0.230 units between nutrient-rich and fasting conditions.^167^ Versions of the biosensor targeted to the cytosol [retaining the nuclear export signal (NES)], nucleus [by removing the NES via XbaI/SalI digestion and ligating with complementary oligonucleotides encoding a C-terminus SV40 nuclear localization signal, or nuclear localization sequence (NLS)], mitochondria (by removing the NES via XbaI/SalI digestion and ligating with complementary oligonucleotides encoding a mitochondrial targeting sequence from the last 29 amino acids of the C-terminus of human monoamine oxidase), and lysosomal surfaces (by removing the NES via XbaI/SalI digestion and ligating with complementary oligonucleotides encoding a 15-amino-acid C-terminus CAAX lysosomal targeting sequence from the Rheb protein) were developed, enabling investigations into compartment-specific mTOR signaling. Depolarization in primary cultured hippocampal neurons increased mean BRET intensity, indicating elevated mTORC1 activity in response to circuit activity. In primary neuronal cultures from Fmr1 knockout and Shank3Δ11 mice (models of autism spectrum disorder), AIMTOR showed higher basal mTORC1 signaling than in wild-type neurons. However, unlike wild-type neurons, depolarization did not further increase AIMTOR BRET signals in these mutants, revealing mTORC1 dysfunction in autism spectrum disorders.^167^

#### 3.2.2 TORCAR

mTORC1 activity reporter (TORCAR) is a FRET-based biosensor consisting of full-length 4EBP1, an mTORC1 target protein, linked between a cerulean and YPet FRET pair on its N- and C-terminals, respectively.^168^ Although multiple configurations of FRET pairs were tested during development of TORCAR, the dynamic range appears limited in that NIH-3T3 cells showed only a roughly 15% increase in FRET ratio in response to PDGF stimulation.^168^ TORCAR has yet to be examined *in vivo*.

### 3.3 Phosphatase and Tensin Homolog (PTEN)

PTEN counteracts the PI3K/Akt/mTOR axis to regulate metabolic activity, ensuring balance in biosynthetic processes.^169^ When PTEN is reduced or inactivated, Akt signaling frequently becomes hyperactive, leading to excessive biosynthetic activity and disproportionately elevated energy demands.^169^ Enzymatically inactive PTEN in AD contributes to abnormal tau phosphorylation and Aβ accumulation, intensifying disease progression.^170^^,^^171^ Meanwhile, PD models indicate that PTEN-mediated inhibition of PI3K/Akt signaling detrimentally influences dopaminergic neuron survival, positioning PTEN as a potential target for neuroprotection.^172^^,^^173^ Indeed, in stroke, brain injury, spinal cord injury, and optic nerve crush, suppressing PTEN boosts neuronal survival and fosters axon regeneration, underscoring its therapeutic promise for CNS repair.^174^^–^^176^

#### 3.3.1 G- and R-PTEN

G-PTEN is a FLIM biosensor that consists of PTEN flanked by an N-terminal fusion of eGFP and a C-terminal fusion of the dead-YFP variant sREACh. G-PTEN was optimized to reduce the effects of PTEN overexpression and demonstrates a roughly 10% dynamic range. The red-shifted R-PTEN is similar but consists of N-terminal mCyRFP2 and C-terminal mMaroon1 for multiplexed imaging with other shorter wavelength biosensors. G- and R-PTEN have been used to track PTEN activity in *C. elegans* and mouse neurons *in vivo*. High-resolution *in vivo* imaging of G-PTEN expressed in mouse pyramidal neurons demonstrated increased PTEN activity in response to CRISPR-mediated knockout of insulin-like growth factor 1 receptor and tuberous sclerosis complex 2.^177^ The role of PTEN signaling in neuronal circuit rewiring was directly demonstrated by *in vivo* imaging of G-PTEN in the whisker barrel cortex during a whisker deprivation model. Interestingly, although whisker deprivation led to a roughly 8% increase in G-PTEN FLIM in inhibitory neurons, PTEN activation was actually slightly reduced in excitatory neurons with presumably less circuit activity.^177^

### 3.4 Hypoxia Inducible Factor-1*α* (HIF-1*α*)

HIF-1α signaling senses depletion of oxygen levels and controls mitochondrial function and cellular bioenergetics to maintain cellular function in an anaerobic environment.^178^^,^^179^ Under physiological conditions where oxygen is present, HIF-1α protein is constantly degraded *via* its oxygen-dependent degradation (ODD) domain, being tagged for proteasomal degradation by prolyl-4-hydroxylases (PHD). When oxygen levels are depleted, PHD activity is substrate-limited, allowing HIF-1α protein to build up and upregulate expression of numerous target genes, including glycolytic enzymes, glucose transporters, fatty acid synthesis enzymes, and components required for autophagy.^180^ In AD, HIF-1α counters neuronal loss by a mechanism that upregulates glycolysis and beta-site amyloid precursor protein cleaving enzyme 1 expression, potentially fueling amyloidogenic processing.^181^^,^^182^ Ischemic stroke studies highlight a time-dependent role for HIF-1α, which initially exacerbates cell death but later confers pro-survival benefits.^183^ TBI models reveal that HIF-1α upregulation promotes neuroprotection, partly *via* erythropoietin induction.^184^^,^^185^

#### 3.4.1 ODD-Luc and ODD-GFP

The ODD type of HIF-1α biosensors fuse the ODD domain (amino acids 530-603) from HIF-1α onto a fluorescent or bioluminescent reporter (ODD-GFP and ODD-Luc, respectively). Like endogenous HIF-1α, ODD-GFP increases in protein abundance and thus fluorescence intensity when oxygen levels are depleted.^186^ ODD-GFP has been expressed in *Drosophila* larvae and was able to detect differences in cellular oxygenation states dependent on distance from respiratory tubes.^186^ The ODD-Luc reporter, a bioluminescent fusion of the HIF-1α ODD domain and firefly luciferase, monitors HIF-1α stabilization. In neuroblastoma cells, it shows dose-dependent stabilization within 2 h by PHD inhibitors such as ciclopirox (CPO, targets iron-binding site), 3,4-dihydroxybenzoate, and dimethyloxaloylglycine.^187^ In hypoxia, ODD-Luc activity increases sixfold after 2 h, but only twofold in glucose-free media, indicating glucose dependency. This glucose-dependent HIF-1α stabilization in hypoxia primarily depends on ATP levels, not PHD activity or transcriptional changes.^188^

Signals from HIF-1α biosensors can be further amplified by combining the ODD protein degradation motif with a gene regulatory element that is recognized by HIF-1α under hypoxic conditions. The hypoxia response element (HRE) is recognized by HIF transcriptional complexes when HIF-1α can be accumulated during hypoxia. By expressing ODD-Luc under the transcriptional control of HRE, HRE-ODD-Luc showed a fourfold increase in expression after 16 h of hypoxia.^189^

### 3.5 Nuclear Factor Erythroid 2-Related Factor 2 (Nrf2)

Nrf2 governs cellular redox balance by detecting oxidative or electrophilic stressors. Once activated, Nrf2 relocates to the nucleus, where it stimulates transcriptional programs that enhance antioxidant capacity and protective detoxification, thereby maintaining mitochondrial function and safeguarding ATP production against oxidative damage.^190^^,^^191^ Nrf2 also regulates glucose uptake and increases both NADH and NADPH cytoplasmic levels in neurons.^192^ The Nrf2 pathway has been linked to mechanisms in neurological disorders. In AD and PD, diminished Nrf2 activity correlates with heightened neuroinflammation, and Nrf2 activation reduces inflammatory damage by enhancing autophagy.^193^^,^^194^ In HD, upregulating Nrf2 clears mutant huntingtin aggregates and bolsters mitochondrial function.^195^^,^^196^ In ALS, Nrf2 upregulation preserves neuromuscular junctions and prolongs survival.^197^^,^^198^ Nrf2 overexpression in astrocytes enhances neuroprotection, whereas its neural activation addresses oxidative stress in multiple sclerosis, ischemic stroke, and retinal ganglion cell degeneration.^199^^–^^202^

#### 3.5.1 POINTER

Pericellular oxygen–insensitive Nrf2 total level reporter (POINTER) is a biosensor that uses transcriptional regulation of miniGFP to measure Nrf2 activity. The transcriptional regulatory promoter uses four repeats of antioxidant response element (ARE) sequences from the promoter of NAD(P)H quinone dehydrogenase 1 (NQO1) to drive the expression of miniGFP. POINTER was evaluated in cultured human cerebral microvascular endothelial cells, where it demonstrated clear distinctions in Nrf2 activity between normoxia and physiological hypoxia. The fluorescence intensity of miniGFP correlated strongly with real-time PCR measurements of oxidative stress–related transcripts, including NQO1, heme oxygenase 1, and glutamate–cysteine ligase catalytic subunit, confirming its functional accuracy.^203^

### 3.6 Guidelines for Using These Biosensors in Studies of Neurodegenerative Diseases

The advent of genetically encoded biosensors has dramatically transformed our capability to observe substrate metabolism in neurons with remarkable spatial and temporal precision. When employing these biosensors to monitor substrate metabolism in living animals, several important factors must be considered. First, specificity and responsiveness are crucial. The physiological levels of different metabolic substrates in mammalian cells vary widely. For metabolites present in high abundance (such as glucose, ATP, lactate, and NAD+), which typically range from 0.1 to 5 mM,^102^^,^^204^^,^^205^ biosensors with lower affinity are recommended. For metabolites found in lower concentrations (such as NADH, NADP+, and NADPH), typically ranging from 0.1 to 30  μM,^103^^,^^206^^,^^207^ high-affinity biosensors are preferable. The affinity of each biosensor is listed in Table 1; however, some sensors may have limited practical utility because their binding affinities fall outside the physiological range.

**Table 1:** Genetically encoded fluorescent biosensors for the study of neurodegeneration.

Sensors	Metabolites sensed	Scaffold	Fluorescent protein	Detection mode	Dynamic range (%)	Substrate range *in vitro*	Reference
ATeam1.03	ATP	ε subunit of $F_{0}F_{1}$-ATP synthase, *Bacillus subtilis*	mscCFP/mVenus	FRET	150	∼1−10 mM	27 and 187
ATeam3.01	ATP	ε subunit of $F_{0}F_{1}$-ATP synthase, *Bacillus* *PS3*	mscCFP/mVenus	FRET	100	∼1−100 μM	27
iATPSnFRs	ATP	ε subunit of $F_{0}F_{1}$-ATP synthase, *Bacillus* *PS3*	cp-SFGFP	Fluorescence intensity	190–240	∼10 μM−10 mM	35
Perceval	ATP/ADP	GlnK1, *Methanococcus jannaschii*	cpmVenus	Excitation ratiometric	80	∼0.1−10	39
PercevalHR	ATP/ADP	GlnK1, *Methanococcus jannaschii*	cpmVenus	Excitation ratiometric	300 (RT), 360 (37°C)	∼0.1−100	40
Peredox	NAD+/NADH	T-Rex, *Thermus aquaticus*	cp-T-Sapphire	Fluorescence intensity	150	∼10−100	54
SoNar	NAD+/NADH	T-Rex, *Thermus aquaticus*	cpYFP	Excitation ratiometric	1400 (RT)	∼1−1000	57
FiNAD	NAD+/AXP	T-Rex, *Thermus aquaticus*	cpYFP	Excitation ratiometric	700	∼0.1−100	58
LigA-cpVenus	NAD+	DNA ligase, *E. faecalis*	cpVenus	Excitation ratiometric	100	30 μM−1 mM	
Frex	NADH	Rex, *Bacillus subtilis*	cpYFP	Excitation ratiometric	800	∼0.5−15 μM	
RexYFP	NAD+/NADH	T-Rex, *Thermus aquaticus*	cpYFP	Fluorescence intensity	300	∼0−3	
FLII^12^ Pglu	glucose	MglB, *E. coli* glucose/galactose-binding protein	eCFP/ Citrine	FRET	30–65 (RT)	∼0.1−10 mM	64
Laconic	lactate	LldR, *E.coli*,	mTFP/ Venus	FRET	8, −1120	∼1 μM−10 mM	68,74
eLACCO1.1	L-lactate _extracellular_	cpGFP, TTHA0766, *Thermophilus*	cpGFP	Fluorescence intensity	300–360	∼1 mM−100 mM	80
R-iLACCO1	L-lactate _intracellular_	cpmApple, LldR, *E. coli*	cpmApple	Fluorescence intensity	74, 230, 350	∼1 μM−1 mM	80
Pyronic	Pyruvate	PdhR, *E. coli*	mTFP/Venus	FRET	24 (RT)	∼10 μM−1 mM	68 and 84
PyronicSF	pyruvate	PdhR, *E. coli*,	cpGFP	Excitation ratiometric	480 (typical)	∼0.1−100 mM	86
ABKAR	AMPK, BRSK	AMPK substrate domain, phosphoamino acid–binding domain (FHA1)	CFP/YFP	FRET	39.4 ± 2.1	NA	160
AIMTOR	mTOR	mTOR substrate domain,	Luciferase/ YPET	BRET	∼0.230	NA	167
G and R-PTEN	PTEN	PTEN core	mEGFP/sREACh	Fluorescence Intensity	∼15	NA	177
			mCyRFP2/mMaroon1				
ODD-GFP	HIF-1α	ODD domain (residues 401–603 of HIF-1α), GFP variant	GFP	Fluorescence intensity	NA	NA	189
HRE-luciferase	HRE	Hypoxia response elements (HREs), minimal promoter,	Luciferase	Bioluminescence	200	NA	189
ODD-Luc	HIF-1α	ODD domain, from the HIF-1α protein (residues 401–603),	Luciferase	Bioluminescence	250–500	NA	209
POINTER	Nrf2	Target-binding domain: derived from one protein partner in the PPI, regulatory domain	miniGFP	Fluorescence intensity	NA	NA	203

In addition, the effect of pH on sensor performance is a critical consideration. Many cpFP-based biosensors are sensitive to pH fluctuations, which can affect their fluorescence readouts. Furthermore, significant glycolytic rate changes can alter intracellular pH, potentially interfering with sensor accuracy. To ensure accurate measurements, it may be necessary to correct for pH effects using a pH control sensor. Last, when combining different biosensors within the same cell *in vivo*, the fluorescence emission spectra of each sensor must be considered to avoid overlap and ensure a clear, distinct readout.

Overall, genetically encoded fluorescent sensors excel in providing detailed *in situ* measurements, and integrating these tools with metabolomics and metabolic flux analysis can enhance our understanding of global metabolic profiles and networks. The ability to resolve metabolic dynamics within targeted cellular populations and at subcellular resolution makes biosensors extremely powerful tools for the investigation of neurodegeneration. However, as a field, we have been relatively slow to apply these tools, which has perhaps limited the development of improved next-generation sensors in the vein of the extremely optimized GECI family. We believe that stronger collaborative efforts between the neuroscience and biosensor design fields are needed to accelerate progress in this research area.

## Data Availability

Data sharing is not applicable to this article as no new data were created or analyzed.
